# The Endocannabinoid System, Cannabinoids, and Pain

**DOI:** 10.5041/RMMJ.10129

**Published:** 2013-10-29

**Authors:** Perry G. Fine, Mark J. Rosenfeld

**Affiliations:** 1 Professor of Anesthesiology, Pain Research and Management Centers, Department of Anesthesiology, School of Medicine, University of Utah, Salt Lake City, Utah, USA; and; 2 Chief Executive Officer, ISA Scientific, Draper, Utah, USA

**Keywords:** Cannabinoids, cannabinoid receptors, chronic pain, endocannabinoid system, phytocannabinoids

## Abstract

The endocannabinoid system is involved in a host of homeostatic and physiologic functions, including modulation of pain and inflammation. The specific roles of currently identified endocannabinoids that act as ligands at endogenous cannabinoid receptors within the central nervous system (primarily but not exclusively CB_1_ receptors) and in the periphery (primarily but not exclusively CB_2_ receptors) are only partially elucidated, but they do exert an influence on nociception. Exogenous plant-based cannabinoids (phytocannabinoids) and chemically related compounds, like the terpenes, commonly found in many foods, have been found to exert significant analgesic effects in various chronic pain conditions. Currently, the use of Δ9-tetrahydrocannabinol is limited by its psychoactive effects and predominant delivery route (smoking), as well as regulatory or legal constraints. However, other phytocannabinoids in combination, especially cannabidiol and β-caryophyllene, delivered by the oral route appear to be promising candidates for the treatment of chronic pain due to their high safety and low adverse effects profiles. This review will provide the reader with the foundational basic and clinical science linking the endocannabinoid system and the phytocannabinoids with their potentially therapeutic role in the management of chronic pain.

Pain is an unpleasant, commonly occurring, and universal human experience; it is also a very complex phenomenon. The experience of pain and the resultant emotional state depends as much or perhaps more on the contextual circumstances (how, when, where, and why) of the pain-inciting event as the intensity of the noxious stimulus. And a seemingly similar pain-producing event may be experienced (and communicated) quite differently from person to person, situation to situation, and among various cultures. The neurophysiology of acute pain due to a brief single noxious event is best understood. The nociceptive components of the peripheral and central nervous systems are highly refined to signal warnings of potential or actual tissue damage; reflex and conscious responses are usually adaptive for self-protection. Fortunately, most occurrences of pain are self-limited, resolving quickly with discontinuation of the noxious stimulus or in tandem with tissue healing or resolution of the insult to somatic or visceral structures.

But pain that continues relentlessly due to on-going nociceptive stimulation from unresolved disease (nociceptive pain) or pathophysiological changes within the nervous system (neuropathic pain) serves little purpose. In contrast to acute pain, unresolved pain leads to subliminal and conscious reflex responses that are often maladaptive (Figure 1).^1^ It imparts a tremendous burden on the pain sufferer’s health, social interactions, occupational performance, emotional state, and finances. In turn, chronic pain incurs a significant direct and indirect financial toll on society (Figure 2). In evaluating the prevalence and impact of pain, a recent report by the National Academy of Sciences’ Institute of Medicine concluded that pain-related medical services and loss of productivity cost the United States economy close to one trillion US dollars annually when pain-related costs associated with patients in long-term care and within the military are included.^2^

**Figure 1:**
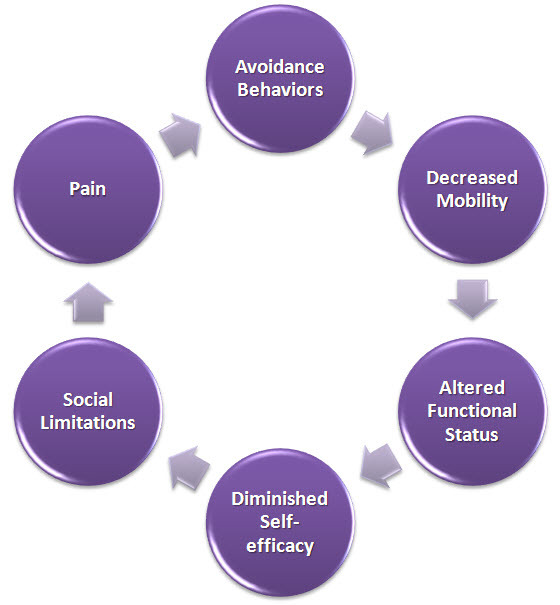
**The Vicious Cycle of Pain.**

**Figure 2:**
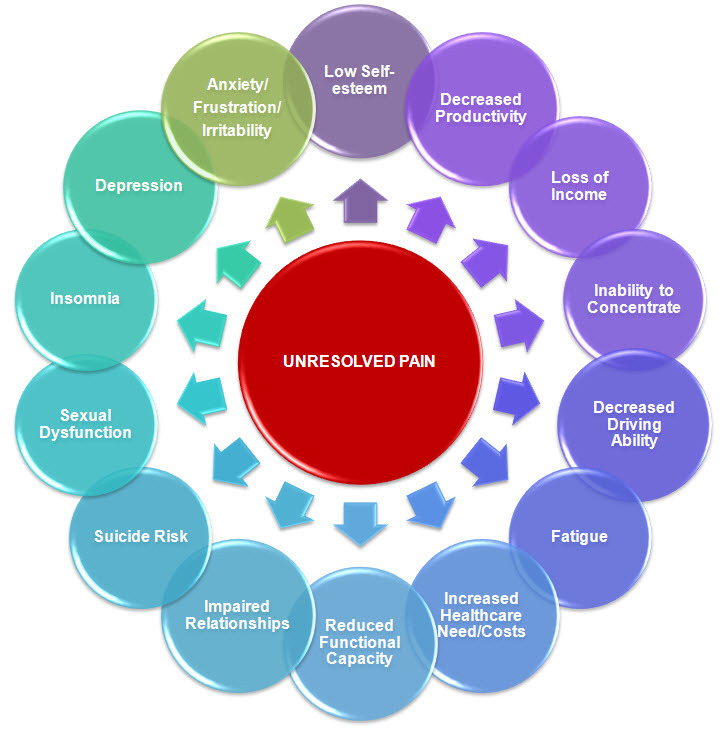
**Consequences of Unresolved Pain.**

The prevalence of persistent, debilitating pain is increasing, commensurate with advances in trauma care that allow survival after serious injury and medical care that slow disease progression, or transform imminently life-threatening diseases into chronic progressive conditions, or provide a cure but with residual morbidity. Similarly, the growing proportion of older individuals in economically developed and developing nations, and the propensity to develop chronic pain-producing conditions with advancing age (e.g. osteoarthritis, degenerative spine disease, vasculopathy, diabetes mellitus, and cancer), is leading to a high prevalence of chronic pain worldwide.

Unfortunately, currently available analgesic medications and pain-modulating procedures are severely limited by combinations of low efficacy, excessive toxicity/risk/safety concerns, insufficient access to care, or unbearable cost. In patients with chronic pain, and especially neuropathic pain, “success” is measured in small increments of improvement among limited numbers of patients. In randomized clinical trials of analgesics for neuropathic pain, no more than half of patients experience clinically meaningful pain relief from pharmacotherapy.^3^ More effective and universally available means to prevent and treat chronic pain are needed, regardless of the primary or inciting cause. Against this background of extraordinary need, this paper will provide an overview of the developing basic and clinical science of cannabinoid pharmacology, and the potential therapeutic value of cannabinoids for chronic pain management.^4^,^5^

The first portion of this article presents a very basic review of the pharmacology of the cannabinoids and endocannabinoid receptor system, drawing both from animal and human models.^6^ Although cannabinoids have putative therapeutic effects in a wide variety of clinical conditions, some of which (e.g. diabetes) are associated with chronic painful conditions, the focus herein is on the effect of cannabinoids on pain rather than on other pathophysiological states. This introduction will pave the way to insight and understanding of the potential role of this class of agents in pain control. Other than to understand basic mechanisms and to formulate hypotheses of safety and efficacy, experience has shown us that animal or human experimental pain investigations poorly predict responses to analgesic therapies in “real life” situations. From this perspective, the second part of this review focuses on pain relief in the clinical setting, and only the human experience will be described.

Extensive research and prolonged exposure to cannabinoids both in animals and humans have addressed important questions about safety. Cannabinoids have a very high therapeutic index. In fact, it is virtually unlimited insofar as fatalities have not been reported directly related to the toxicity of any cannabinoid, even with extremely high dosing. However, there are potentially severe cognitive, psychotomimetic, and substance abuse-related adverse effects associated with Δ9-tetrahydrocannabinol (THC) exposure that must be taken seriously, especially in young or cannabis-naïve patients.^7^–^17^

In medicinal use, these adverse effects may be prevented or mitigated by avoiding THC entirely in favor of other non-psychoactive cannabinoids.^18^ For instance, prolonged exposure to the non-psychoactive phytocannabinoid, cannabidiol (CBD), at doses of 3–4 mg/kg/d, both in human volunteers and those with epilepsy, revealed no adverse effects or evidence of toxicity.^19^ However, adequate precaution must be taken when CBD is used in conjunction with many other drugs due to its inhibition of several cytochrome P450 isoenzymes, including CYP1A2, CYP2B6, CYP2C9, CYP2D6, and CYP3A4. This is especially important in the management of chronic pain, since many conventionally used analgesics (opioids and non-opioids) are metabolized via these pathways (most notable CYP2D6 and CYP3A4).^20^ Therefore, the key relevant clinical issues for practitioners dealing with populations of patients in pain have to do with questions about effects of specific cannabinoids, their various modes of delivery and absorption, potential indications, and their respective risks and tolerability.^21^

Based on relatively new but limited scientifically based literature, it is now only possible to speculate about mechanisms of action and what the future may hold for phytocannabinoids as effective analgesics across the vast and varied cohorts of people living with chronic pain. With that in mind, this review will proceed with a summary of what is known about different cannabinoid congeners on various types of pain (efficacy and tolerability) and the putative role of commonly available “generally regarded as safe” (GRAS) ingredients that may enhance the effectiveness of certain phytocannabinoids.

## CANNABIS AND CANNABINOIDS: PAST TO PRESENT

Cannabinoid refers to a pharmacological class of about 60 naturally occurring compounds (phytocannabinoids) found in plants of the genus *Cannabis* (i.e. marijuana and hemp) and structurally related synthetic analogues (e.g. Δ3,4-tetrahydrocannabinol and HU-210, which is 100–800 times more potent psychoactively than natural THC^22^). This classification has been generalized to include a wide range of exogenous and endogenously produced compounds that exhibit similar pharmacodynamic properties as the phytocannabinoids or demonstrate activity at the same receptor binding sites. *Cannabis sativa* has two subspecies, *indica* and *sativa*. A variety of the former, hemp, has industrially and nutritionally useful qualities. Hemp has a very low amount of the psychoactive constituent Δ9-tetrahydrocannabinol (THC) but higher quantities of cannabidiol (CBD) which may offer a range of medicinal benefits without the cognitive effects and abuse potential associated with THC.^23^

*Cannabis* has a long and storied social and medicinal history dating back thousands of years.^24^,^25^ Regulations restricting *Cannabis* cultivation and distribution, especially as these pertain to marijuana, have preoccupied governments from China, through India to Europe and Great Britain, and across the Atlantic to the Americas for centuries due to its inherent psychedelic intoxication.^26^ The emotional and cognitive effects of *Cannabis* have mostly been sought for recreational or ritualistic purposes, and are commonly derived from smoking dried plant material or its concentrated oily derivative, hashish. Even though hemp has minimal potential psychoactivity, it is nonetheless subjected to the same restrictions as marijuana in many jurisdictions.

Only recently have we gleaned scientific insight into several of the pharmacologically distinct cannabinoids and their effects at specific receptors within various animals and humans. In 1997 both the United States National Institutes of Health and the British Medical Association released reports on the potential therapeutic uses of *Cannabis* and cannabinoids. Notwithstanding the momentous breakthrough represented by these reports in support of the potential value of cannabinoids for medical use, the health hazards of smoking coupled with the cognitive-behavioral effects of *Cannabis* have created political and regulatory obstacles worldwide, with regard to evaluating cannabinoids as medicines and mainstream health care professionals’ acceptance of *Cannabis* as a legitimate therapeutic agent. Fortunately, as the sciences of drug delivery and cannabinoid pharmacology have progressed in recent years, there are rapidly evolving technologies that will facilitate or enhance the medically indicated use of this pharmacological class of agents while overcoming the barriers imposed by unwanted or harmful psychoactive effects of *Cannabis* and smoking it as the only effective way to obtain adequate blood levels of cannabinoids.^27^

The potential value of the cannabinoids for medicinal purposes arose from the discovery^28^ and later cloning of endogenous cannabinoid receptors.^29^,^30^ The two major receptor types, CB_1_ (mostly in the central nervous system) and CB_2_ (mostly in peripheral tissues), are differentiated by their physiological actions and locations within the body. These are members of the seven transmembrane G-protein coupled receptor superfamily which comprise the binding sites for almost half of all contemporary drugs.^31^

## THE ENDOCANNABINOID SYSTEM: RECEPTORS AND ENDOGENOUS RECEPTOR LIGANDS

The endogenous cannabinoid system has been described as “an ancient lipid signaling network which in mammals modulates neuronal functions, inflammatory processes, and is involved in the etiology of certain human lifestyle diseases, such as Crohn’s disease, atherosclerosis and osteoarthritis. The system is able to downregulate stress-related signals that lead to chronic inflammation and certain types of pain, but it is also involved in causing inflammation-associated symptoms, depending on the physiological context.”^32^

### CB_1_ Receptors

The CB_1_ receptor has been cloned from humans.^33^ Activation of CB_1_ receptors leads to dose-dependent and stereo-selective inhibition of adenylate cyclase activity, thus affecting memory, perception, and movement. The CB_1_ receptor appears to be responsible for the mood-enhancing effects of *Cannabis* as well as negative, dysphoria-inducing, and frank psychotomimetic effects in susceptible individuals.

CB_1_ receptor distribution has been well characterized in the human brain.^34^ The receptors are expressed in high abundance in the hippocampus and associational cortical regions, the cerebellum, and the basal ganglia. This widespread distribution in the brain matches well with the known pharmacodynamic effects of cannabinoids. In contrast, binding is sparse or absent from the brain stem, medulla, and thalamus. The paucity of CB_1_ receptors in these areas helps explain the absence of life-threatening effects on vital physiological functions associated with extremely high doses of cannabinoids.

Besides the brain, the CB_1_ receptor occurs in the testis, and presynaptically on sympathetic nerve terminals.^35^ CB_1_ receptor mRNA has been identified in the adrenal gland, heart, lung, prostrate, bone marrow, thymus, and tonsils.^36^,^37^

### CB_2_ Receptors

Although CB_1_ and CB_2_ receptors share considerable structural similarities, their distribution and activity diverge. Among other actions, including pain modulation, CB_2_ receptors are thought to serve an important role in immune function and inflammation.^38^ There is ample evidence that CB_2_ receptor activation reduces nociception in a variety of preclinical models, including those involving tactile and thermal allodynia, mechanical and thermal hyperalgesia, and writhing.^39^ With regard to their role in modulating neuropathic pain, the presence of CB_2_ receptors on microglia within the nervous system may explain the putative benefits of cannabinoids in reducing cytokine-mediated neuroinflammation.

CB_1_ and CB_2_ receptors inhibit adenylate cyclase via interactions at the G-protein complex. However, their activation and consequent inhibition of various ion channels differs.^40^ The key point is that differential binding of CB_1_ or CB_2_ receptors, either separately or in combination by their respective endogenous or exogenous ligands, leads to varied physiological effects (Table 1), mediated via several neurotransmitters, including acetylcholine, glutamate, and dopamine.

**Table 1:** Physiological Actions Mediated by Activation or Inhibition of Cannabinoid Receptors.

Physiological Actions
1. Antinociception
2. Cognition and memory
3. Locomotor activity
4. Endocrine functions
5. Temperature control and heart rate
6. Nausea and vomiting
7. Intraocular pressure
8. Inflammation
9. Immune recognition and antitumor effects

## ENDOGENOUS CANNABINOIDS AND NOCICEPTION

**Figure 3:**
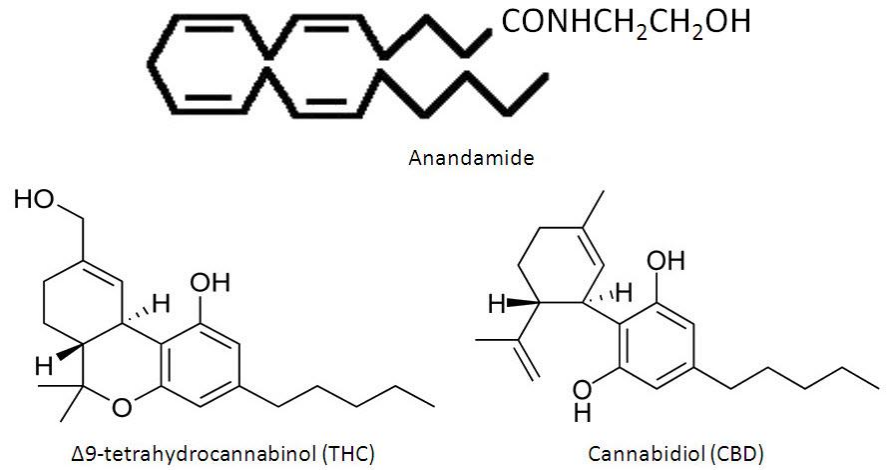
**Chemical Structures of Anandamide, Δ9-Tetrahydrocannabinol, and Cannabidiol.**

The first compound to be identified as an endogenous cannabinoid receptor ligand was given the name anandamide, after the Sanskrit word for “bliss.” Anandamide (Figure 3) bears no chemical resemblance to the aromatic phytocannabinoids such as THC and CBD, but rather is an arachidonic acid derivative.^41^ Several other endogenously generated moieties have been identified that bind to cannabinoid receptors (collectively known as endocannabinoids), but their roles in homeostatic functions and in disease states remain poorly defined. The physiologic role of anandamide continues to be actively explored, having been identified in central and peripheral tissues of man.^42^

It appears that the endocannabinoid system is intimately involved in tissue healing in the face of inflammatory conditions, correlating clinically with prevention and treatment of inflammation-mediated pain.^43^ With regard to potential pain-modulating activity, anandamide has been shown to be a full agonist at vanilloid (TRPV_1_) receptors and may play a modulating role at other transient receptor potential (TRP) receptor types.^44^ Anandamide is reported to produce effects similar to THC at CB_1_ receptors, via G-protein coupled inhibition of adenylate cyclase. These effects include antinociception, hypomotility, and reduced memory.^45^

However, there appear to be distinct differences between anandamide and other cannabinoids with respect to their antinociceptive properties and other physiological effects which vary as a function of route of administration. It is not known whether anandamide acts at the same sites as phytocannabinoids to produce antinociception. The behavioral effects of THC and anandamide after administration suggest that they act, at least in part, in the brain and/or spinal cord. These studies suggest that anandamide is less potent and has a shorter duration of action than THC.^46^

Studies have demonstrated that antinociceptive effects of cannabinoids are mediated through mechanisms distinct from those responsible for other behavioral effects. For instance, THC has additive analgesic efficacy with kappa opioid receptor agonists. This effect is blocked by kappa antagonism, but opioid receptor antagonism does not alter the psychoactive effects of THC.^47^ Investigations into the endogenous cannabinoids and their effector sites (including CB_1_ and CB_2_ along with other non-cannabinoid receptors) have exploded in recent years, and insights reveal this area of pharmacology to be highly complex and dynamic. For instance, there is mounting evidence that endogenous and exogenous cannabinoids exert some influence on opioid, 5HT_3_, and N-methyl-d-aspartate receptors. These interactions suggest a role for endocannabinoids in homeostatic pain modulation (antinociception), thus their use as exogenous agents in pain management.^48^

Most recently, Thiago et al.^49^ provided evidence that the cannabinoid agonists anandamide and N-palmitoyl-ethanolamine (PEA) induce peripheral antinociception activating CB_1_ and CB_2_ receptors, respectively, stimulating the endogenous noradrenergic pathway which in turn activates peripheral adrenoreceptors, inducing antinociception. Other studies have demonstrated the expression of functional CB_2_ receptors in areas of human dorsal root ganglion (DRG) sensory neurons. CB_2_ receptor expression also has been demonstrated in the spinal cord as well as in other brain regions particularly relevant for nociceptive integration.^50^–^52^

These findings implicate CB_2_ receptors in the analgesic effects produced by CB_2_ agonists.^53^,^54^ Other evidence for the involvement of the endocannabinoid system in peripherally mediated pain control includes the finding that CB_2_ receptor agonists can evoke analgesia by triggering the release of beta-endorphin in response to the stimulation of CB_2_ receptors expressed in human keratinocytes.^55^ Many other studies have linked cannabinoid and opioid effects through primary receptor interactivity as well as downstream second messenger effects. From a clinical standpoint, this may provide an opportunity for therapeutic synergy.^56^

The role of CB_2_ receptors in antinociception has been demonstrated in inflammatory and neuropathic pain models. Investigations involving carrageenan-induced inflammatory pain in rodents demonstrate that activation of CB_2_ receptors by CB_2_-selective agonists suppresses neuronal activity in the dorsal horn via reduction in C-fiber activity and wind-up involving wide dynamic range (WDR) neurons.^57^,^58^ The involvement of cannabinoid receptors in modulating pain has been supported further by findings that there are increases in peripheral CB_2_ receptor protein or mRNA in inflamed tissues and in the dorsal root ganglion in neuropathic states.^59^–^61^ Data from studies investigating viscerally induced pain due to colorectal distension indicate that peripheral CB_1_ receptors mediate the analgesic effects of cannabinoids on visceral pain from the gastrointestinal tract.^62^

It may now be concluded that cannabinoids play a role in endogenous (homeostatic) modulation of nociception, and that exogenous cannabinoids potentially offer some degree of analgesia in various pain states.^63^ With this foundation to build upon, the proceeding section will explore the role of cannabinoids in clinical pain relief in humans. Much has been learned since a decade ago when there was significant doubt about translating research findings linking cannabinoids to antinociception from “bench to bedside.”^64^ There are now methodically sound studies that may lead to important therapeutic advances for people living with pain.

## CANNABINOIDS AND THE MANAGEMENT OF PAIN

Evidence continues to accumulate suggesting that cannabinoids can impact normal inhibitory pathways and pathophysiological processes influencing nociception in humans.^59^,^65^ When cannabinoids do have an analgesic effect, it is more likely to occur in hyperalgesic and inflammatory states.^66^ Clinical trials lasting from days to months, involving more than 1,000 patients, have shown efficacy in different categories of chronic pain conditions (Table 2), but the vast majority of controlled trials have involved patients with chronic neuropathic pain.^67^–^78^

**Table 2:** Positive therapeutic trials treating chronic painful conditions with cannabinoids.^67^–^78^

**Type of Pain and Condition (if described)**	**Number of Subjects**	**Cannabinoid Type, Preparation**	**Dosage and Route**	**Treatment Duration**	**Study Design**	**Results**	**Author, Reference #**
**NEUROPATHIC PAIN**							
HIV	50	Marijuana	3.56% THC, smoked 3 tid	5d	RCT	Significant pain reduction in active treatment group	Abrams et al.^67^
Chronic NP pain	34	THC+CBD 1:1	Oral mucosal, variable dose	12 wks	RCT	Positive pain relief (not otherwise specified)	Notcutt et al.^68^
Chronic NP pain	21	CT-3 (THC analogue)	Oral, 20 mg bid × 4d, then 40 mg bid × 3d	7d	RCT cross-over	Significant decrease in hyperalgesia, allodynia, and VAS pain intensity scores	Karst etal.^69^
Multiple sclerosis	630	THC cannabis extract	Oral, variable dose	15 wks, with 52 wks continuation	RCT	Statistically significant reduction in pain scores and clinically meaningful sense of improvement	Zajicek et al.^70^
Multiple sclerosis	24	Dronabinol	Oral, 10 mg	3 wks	RCT cross-over	Significant pain reduction with active treatment	Svendsen et al.^71^
Multiple sclerosis	137	THC+CBD 1:1 (Sativex™)	Oral mucosal, variable dose	10 wks controlled trial followed by 52 wks open label	RCT and open label	Significant pain reduction with active treatment; continued pain relief in about half of long-term use patients	Wade et al.^72^
Multiple sclerosis	66	THC+CBD 1:1 (Sativex™)	Oral mucosal, variable dose	4 wks	RCT	Significant pain reduction with active treatment	Rog etal.^73^
Chronic NP pain conditions	24 total: MS−18; BPI−1 SCI−4; PLP−1	THC+CBD 1:1	Oral mucosal, variable dose	2 wks	RCT cross-over	Significant pain reduction with active treatment	Wade et al.^74^
Brachial plexus injury	48	THC-CBD 1:1 (Sativex™)) vs THC vs placebo spray	Oral mucosal, variable dose	3 × 2-week treatment periods	RCT cross-over	Significant pain reduction with both active treatments	Berman et al.^75^
Peripheral NP pain	125	THC+CBD 1:1 (Sativex™)	Oral mucosal, variable dose	5 wks controlled trial followed by 52 wks extension	RCT	Significant pain reduction with active treatment	Nurmikko etal.^76^
**INFLAMMATORY PAIN**							
Rheumatoid arthritis	58	THC+CBD 1:1 (Sativex™)	Oral mucosal, variable dose	5 wks	RCT	Significant pain reduction in active treatment group both at rest and with movement	Blake et al.^77^
Acute pancreatitis	Not specified	HU-210 (synthetic CB, and CB_2_ agonist)	Oral	Not specified	Not specified	Significant pain reduction	Michalski et al.^78^

bid, twice a day; BPI, brachial plexus injury; CBD, cannabidiol; d, days; HIV, human immunodeficiency virus; MS, multiple sclerosis; NP, neuropathic pain; PLP, phantom limb pain; RCT, randomized controlled trial; SCI, spinal cord injury; THC, delta-9-tetrahydrocannabinol; tid, three times a day; wks, weeks.

When cannabinoids lead to a reported reduction in pain, it remains unclear where the effects are triggered, or which aspect of the pain experience is most affected and under what circumstances. As well, different cannabinoids may lead to mechanistically different pain-relieving effects. For instance, a recent study of functional brain imaging in human volunteers investigated the means by which THC may influence pain resulting from capsaicin-induced hyperalgesia. The study results suggest that “peripheral mechanisms alone cannot account for the dissociative effects of THC on the pain that was observed. Instead, the data reveal that amygdala activity contributes to inter-individual response to cannabinoid analgesia, and suggest that dissociative effects of THC in the brain are relevant to pain relief in humans.”^79^ In other words, cannabinoids, and THC in particular, may have differential effects on the sensory (e.g. intensity; quality) versus affective (e.g. unpleasantness; suffering) components of pain.

The two best-studied cannabinoids implicated as having potential analgesic properties are THC and CBD (Figure 3). THC was first isolated from *Cannabis* by Raphael Mechoulam and colleagues in 1964 at the Hebrew University of Jerusalem, and they identified it as the major psychoactive component of *Cannabis*, with preferential binding at CB_1_ receptors.^80^ Synthetic forms of THC, like dronabinol and nabilone, are commercially available in several countries, and are considered controlled substances. These have indications for treating anorexia in AIDS patients and as a therapy for intractable nausea and vomiting during cancer chemotherapy. In a wide range of oral doses, dronabinol, which is chemically identical to the THC extracted from plants, has not demonstrated significant pain relief in several naturally occurring and experimental pain conditions.^81^–^83^ In contrast, nabilone, which is chemically similar to THC but not identical,^84^ has demonstrated modest efficacy in fibromyalgia^85^ but with dose-limiting adverse effects. Its use has led to paradoxical increases in pain in the postoperative setting.^86^

Cannabidiol is a major constituent of *Cannabis*. It has virtually no psychoactivity compared against THC.^87^ Cannabidiol has low affinity for both cannabinoid CB_1_ and CB_2_ receptors. Limited pharmacodynamic effects due to relatively weak receptor binding (low affinity) may be overcome with higher doses of agonist. Whereas the dose-limiting factor with THC resides in the highly variable propensity among individuals to experience and tolerate negative affective, cognitive, and psychotomimetic effects, the ability of cannabidiol to behave as a CB_1_ receptor inverse agonist may contribute to its documented mitigating action on THC psychotomimetic effects. More recently it has been postulated that cannabidiol may exert its effects via inhibition of anandamide deactivation or otherwise enhancing anandamide signaling.^88^

Cannabidiol agonist activity at CB_2_ receptors seems to account for its anti-inflammatory properties and both primary and secondary influences on pain.^89^,^90^ As well, memory impairments associated with THC are not apparent with CBD, and, when combined, CBD reduces the negative impact of THC on memory. This mitigating effect also has been attributed to the inverse agonist effect at CB_1_ receptors by CBD. Anxiolytic effects of CBD may also be attributed to its agonist effect at the 5-HT_1A_ receptor.^91^

A pharmaceutical combination product of THC and CBD now exists as an oral spray consisting of 27 mg Δ9-tetrahydrocannabinol and 25 mg cannabidiol per mL (100 microliters per administered dose; i.e. 2.7 mg THC and 2.5 mg CBD), extracted from *Cannabis sativa* L. This formulation is approved in Canada, New Zealand, Israel, and several European countries (and possibly seeking US FDA approval in 2013) for the management of spasticity in multiple sclerosis (MS). There are several on-going trials on its efficacy in treating MS-related pain.^92^ Investigations of the therapeutic value of THC and THC–CBD via oral mucosal delivery in the treatment of various other neuropathic pain conditions show promising albeit modest results.^5^,^73^,^75^,^93^ The limited efficacy is likely due to the relatively low dose of this combination of cannabinoids. It is important to note that the dose-limiting factor is how much THC may be tolerated. With higher doses via smoking marijuana or inhaling vaporized *Cannabis*, hyperalgesic and cognitive effects become more pronounced and problematic, especially in cannabis-naïve individuals.^94^–^98^ Beyond these trials involving CBD and THC, comparative or head-to-head studies of individual cannabinoids or various cannabinoid combinations and routes of administration evaluating clinical outcomes are lacking.

## CANCER PAIN

The therapeutic role of cannabinoids in cancer treatment, in terms of effects on tumor cells and on cancer pain, is of great interest. Correlations have been found between cannabinoid receptor levels and endocannabinoid activity and cancer severity, pain intensity, and survival.^99^

For treating refractory cancer-related pain, there is mounting evidence that cannabinoids may be a useful addition to current analgesic treatments. However, to realize the full potential of cannabinoids suggested by preclinical data, it is likely that peripheral CB_1_ or CB_2_ receptors or modulation of endocannabinoids will have to be targeted to achieve analgesia without dose-limiting side effects.^100^, ^101^ So far, studies of the efficacy of CBD in cancer pain (as well as in neuropathic pain) have used insufficient doses of CBD (alone or in combination with THC) to determine efficacy.^102^ Part of this insufficiency may be due to the poor bioavailability of cannabinoids.^103^

## COMBINING PHYTOCANNABINOIDS AND TERPENES: THE ENTOURAGE EFFECT

The entourage effect is the term used to describe enhancement of efficacy, with related improvement in overall therapeutic effectiveness, derived from combining phytocannabinoids and other plant-derived molecules.^104^ Besides CBD, phytocannabinoids that have been identified as exerting clinically useful effects without psychoactivity include tetrahydrocannabivarin, cannabigerol, and cannabichromene. Innovative conventional plant breeding has been yielding *Cannabis* chemotypes expressing high titers of each component for future study.

A chemical class known as the terpenes shares a precursor molecule with phytocannabinoids; they are all flavor and fragrance components common to human diets. Terpenes have been designated “generally recognized as safe” (GRAS) by the US Food and Drug Administration and other regulatory agencies. *Cannabis*-derived terpenes include limonene, myrcene, α-pinene, linalool, β-caryophyllene, caryophyllene oxide, nerolidol, and phytol.^105^ These terpenes are also found in other plants.

Terpenes are quite potent and affect animal and even human behavior when inhaled in very low concentrations. They display unique therapeutic effects that may contribute meaningfully to the entourage effects of *Cannabis*-based medicinal extracts. Of particular interest are the phytocannabinoid–terpene interactions that could produce synergy with respect to treatment of pain and inflammation. Phytocannabinoid–terpene synergy increases the likelihood that an extensive pipeline of new therapeutic products is possible from this age-old plant.

The synergistic contributions of cannabidiol to *Cannabis* pharmacology—and specifically analgesia—have been scientifically demonstrated. Preclinical and clinical data indicate that cannabinoids administered together are more effective at ameliorating neuropathic pain than the use of a single agent.^104^,^106^

The terpene β-caryophyllene is found in a number of commonly available plants, including black pepper, cinnamon, clove, and other spices. It selectively binds to the CB_2_ receptor at nanomolar concentrations and acts as a full agonist. β-Caryophyllene and cannabidiol occur abundantly in *Cannabis sativa.* So this plant species produces at least two entirely different chemical substances able to target CB_2_ receptors differentially. While studies on the pharmacokinetics of β-caryophyllene are still on-going, it is already clear that this terpene is readily bioavailable. Unlike many polyphenolic natural products, it is not metabolized immediately but shows a Tmax >1 h after one single oral administration. Orally administered β-caryophyllene (<5 mg·kg^−1^) produces strong anti-inflammatory and analgesic effects in wild-type mice but not in CB_2_ receptor knock-out mice, which is a clear indication that it may be a functional CB_2_ ligand.^107^

On-going studies show that β-caryophyllene is effective at reducing neuropathic pain in a CB_2_ receptor-dependent manner.^108^ Like other CB_2_ ligands β-caryophylleneinhibits the pathways triggered by activation of the toll-like receptor complex CD14/TLR4/MD2, which typically leads to the expression of pro-inflammatory cytokines (e.g. IL-1 beta, IL-6, IL-8, and TNF alpha) and promotes a Th1 immune response that plays a critical role in neuroinflammation, sensitization, and pain.^109^ Therefore, the FDA-approved food additive β-caryophyllene seems an attractive candidate for clinical trials targeting the CB_2_ receptor. Indeed, in cases of intractable or difficult-to-control pain, combination therapy with small doses of opioid and non-psychoactive cannabinoid receptor agonists may be an alternative way to circumvent the undesirable side effects of opioids yet obtain far greater analgesic efficacy than achieved with cannabinoids alone.^56^,^110^

## ADDITIONAL PAIN-RELATED THERAPEUTIC BENEFITS OF CANNABINOIDS

Cannabinoids may have another therapeutic benefit in managing chronic pain, with regard to sleep. Not only does normalized sleep improve pain relief and mood disorders associated with both poor pain control and poor sleep patterns, but there is significant risk of sleep-disordered breathing associated with central nervous system (CNS) drugs used to treat chronic pain.^111^ Opioid analgesics are most problematic, especially if combined with other CNS depressants such as benzodiazepines. It has been reported that cannabinoids suppress sleep-related apnea. This is an important area for further research and clinical application both in sleep and pain medicine.^112^

## CONCLUSIONS

The phytocannabinoids have efficacy in the treatment of various chronic pain conditions with greatest promise as a therapeutic adjunct in treating peripheral and central neuropathic pain and inflammation-mediated chronic pain. However, the smoked route of administration and the psychoactivity of THC—with associated concerns about abuse and long-term cognitive adverse effects—continue to pose serious and significant barriers to obtaining benefit from *Cannabis* among most patients and acceptability among health care professionals and regulatory agencies.

A formidable barrier to oral bioavailability resides in the pharmacokinetics of naturally occurring and synthetic cannabinoids, but relatively slow elimination may provide clinical utility through prolonged duration of therapeutic effects once these agents gain entry into the systemic circulation. The phytocannabinoids are metabolized rapidly in the liver, undergoing extensive hepatic first-pass metabolism.^113^ Elimination of oral cannabinoids from plasma is biphasic with an initial half-life of approximately 4 hours, and the terminal elimination half-lives are of the order of 24 to 36 hours or longer. Cannabinoids are distributed throughout the body; they are highly lipid-soluble and accumulate in fatty tissue. The release of cannabinoids from fatty tissue is responsible for the prolonged terminal elimination half-life.^114^–^116^

Putting these pharmacologic, clinical, and societal issues together, the direction for the future resides in the development of orally administered, highly bioavailable, non-psychoactive phytocannabinoid products that also take advantage of the entourage effect, to provide the millions of people living with debilitating pain a comparatively safe and effective form of relief.

## Abbreviations:

CB_1_ and CB_2_: cannabinoid receptors 1 and 2, respectively;
CBD: cannabidiol;
THC: delta-9-tetrahydrocannabinol.
